# Monostotic Paget's Disease Involving the Scapula Encountered Incidentally on a Trans-arterial Aortic Valve Implantation Scan

**DOI:** 10.7759/cureus.31666

**Published:** 2022-11-19

**Authors:** Suhail Khan, Cleofina Furtado, Walid Al-Deeb

**Affiliations:** 1 Department of Diagnostic and Interventional Radiology, University Hospitals of North Midlands NHS Trust, Stoke-on-Trent, GBR

**Keywords:** transarterial aortic valve implantation (tavi), computed tomography (ct), scapula, monostotic, paget’s disease of bone

## Abstract

Paget's disease of the bone is a chronic bone disorder. However, to our knowledge, only a few cases of Paget's disease with isolated scapular involvement have been documented in the literature. In this case study, we describe an 81-year-old male patient who was incidentally diagnosed with monostotic Paget's disease of the left scapula during a computed tomography scan for the placement of a trans-arterial aortic valve. We discuss how crucial it is to recognise typical imaging appearances in order to prevent unnecessary investigations and interventions.

## Introduction

Paget's disease of the bone (PDB) is a disorder of bone metabolism that occurs in the ageing skeleton [1]. The overgrowth of bone occurs at a single (monostotic PDB) or numerous (polyostotic PDB) locations as a result of an increased rate of bone remodelling [2]. In this condition, the affected bone's integrity is subsequently compromised [3]. PDB affects 2%-7% of the population over 55 years of age in North America and Western Europe [4]. The pelvis, long bones of the lower limbs, spine, and skull are among the most commonly affected locations [5]. It is quite uncommon for the scapula to be involved alone and, to our knowledge, very few reports have been previously described in the literature [6].

Here, we present a rare instance of monostotic PDB limited to the left scapula, detected incidentally during a scan for trans-arterial aortic valve implantation (TAVI).

## Case presentation

An 81-year-old man with a history of hypertension was examined by the cardiology team to determine the cause of his recent increase in breathlessness and decline in exercise tolerance. Clinical investigations revealed severe aortic stenosis. The evaluation of TAVI planning was performed using an electrocardiogram-gated computed tomography (CT) scan.

**Figure 1:**
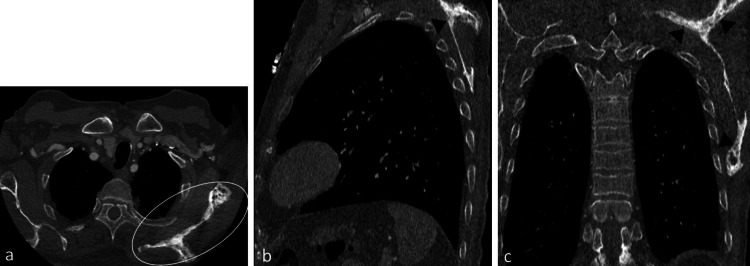
(a) Axial (b) sagittal and (c) coronal reformats of a TAVI scan showing the incidental finding of the expanded cortex of the left scapula with trabecular thickening (white oval in a, and black arrowheads in b and c) TAVI, trans-arterial aortic valve implantation

**Figure 2:**
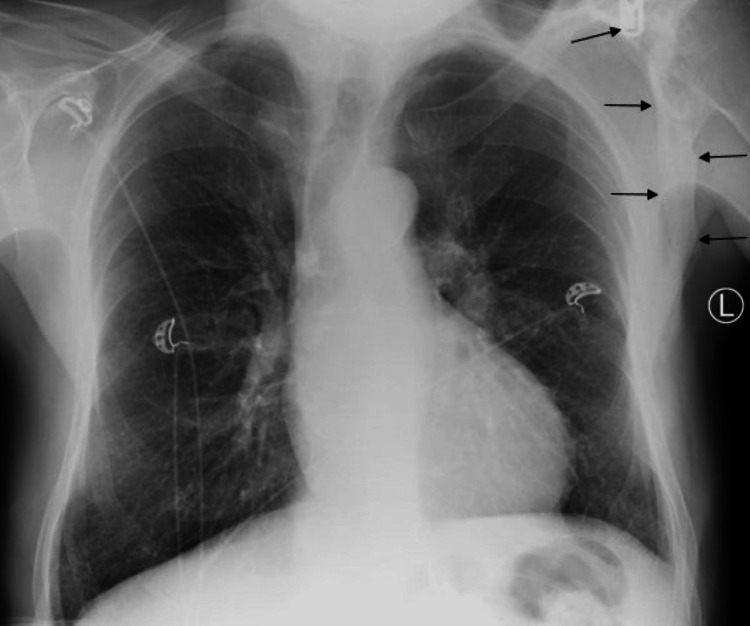
Chest radiograph PA view demonstrating bony enlargement of the left scapula with trabecular thickening (black arrows) PA, posterior anterior

Severe aortic stenosis was seen on the CT scan. In addition, the left scapula demonstrated trabecular thickening, medullary expansion, diffuse cortical thickening, and sclerosis (Figure 1). There was no bony destruction or cortical erosion. The pelvic bones, sternum, ribs, spine, and rib cage were unremarkable. Although the site involved was highly unusual, the imaging characteristics were typical of monostotic Paget's disease. A retrospective evaluation of the chest radiograph obtained the same day prior to CT demonstrated coarsened trabeculae and bony enlargement of the left scapula (Figure 2).

His serum alkaline phosphatase level was 60 IU/L, which falls within the normal range of 30-130 IU/L. Serum calcium and phosphorus levels were found to be normal. An endocrinologist's review resulted in the decision that no additional investigation or treatment was required since there had never been any prior complaints of localised pain or tenderness and the biochemistry was normal. He has continued follow-up imaging for TAVI in accordance with the clinical need.

## Discussion

We describe here a case of monostotic Paget's disease that happened to be discovered in a very unusual location. PDB with the involvement of the scapula is not common, even in the polyostotic pattern (24% of the cases in a study), and we could find only one reported case of monostotic Paget’s involving the scapula in the literature [6,7]. However, the one reported case was of a lung cancer patient and posed a diagnostic challenge to rule out metastatic disease of the bone [6].

Radiographs and CT are usually the diagnostic tests used in Paget’s disease diagnosis, showing characteristic deformities of the bone, with thickened cortices marked by tunnelling and accentuated trabeculae [8]. Plain films obtained earlier in the course of the illness may show a predominantly lytic lesion [9]. Over time, however, there is evidence of an osteoblast response, and the bone thickens and enlarges [10]. In some cases of PDB, there may be dense bone with little evidence of remodelling by biochemical parameters. However, it is important to keep in mind that these stages might exist simultaneously in the same patient and in the same bone [11].

Bone scintigraphy can determine the degree of the disease using whole-body images and can find lesions in places that are challenging for radiography to analyse [7,11]. The bone growth and trabecular thickening in our instance were thought to be characteristic of the mixed stage of Paget's disease with monoostotic involvement. For the detection of bone metastases, positron emission tomography (PET) offers good specificity (97%) and sensitivity (90%) values [12]. However, in our case, there was no history suggestive of any malignant disease that collaborated with CT imaging. Thus, a metastatic deposit was considered highly unlikely, and a PET scan was not performed.

A commonly evaluated serum marker for Paget's is serum alkaline phosphatase, reflecting the elevated rate of bone formation and resorption. However, in the monostotic type of the disease, like in our case, it can be normal [13]. Serum calcium and phosphate levels are expected to be normal, as in this case [14]. Another commonly used marker for bone resorption is urine hydroxyproline. It is a product of collagen and a modified amino acid [15]. However, its measurement poses technical difficulties in collection and measurement and hence was not performed in our case [16].

## Conclusions

Paget's disease of the bone is a well-known entity with a typical distribution, and rarely, the site of involvement can be highly unusual. It is important to recognise the typical findings on commonly performed imaging techniques such as radiographs and CT. Typical imaging findings in correlation with the clinical history of the patient can obviate the need to perform extensive imaging studies and biopsies even when the site of involvement is highly unusual. This would help save hospital resources and avoid causing unnecessary stress to the patient.
